# The Effect of Microstructural Evolution on Mechanical Behavior of Carbon/Carbon Composites After Heat Treatment

**DOI:** 10.3390/ma19081640

**Published:** 2026-04-20

**Authors:** Zhenyu Yuan, Xiao Liu, Yu Yang

**Affiliations:** State Key Laboratory of Powder Metallurgy, Central South University, Changsha 410083, China

**Keywords:** carbon/carbon composites, microstructural evolution, mechanical properties

## Abstract

**Highlights:**

- Under the effect of high-temperature heat treatment, the texture of PyC transforms from SL to RL, while the self-healing of cracks is also promoted.
- The rearrangement of the graphene layers resulted in a weakening of the PyC/fiber interface as well as the bridging of the adjacent fibers.
- The samples with higher texture showed a significant decrease in mechanical properties, but an increase in deformability, which is attributed to increased graphitization and the weakening of the PyC/fiber interface.

**Abstract:**

The effect of microstructural evolution on mechanical behavior of carbon/carbon composites after heat treatment has been investigated. Two kinds of samples, heat-treated at 2300 °C and 2700 °C, were used in the current study. As the heat treatment temperature is 2700 °C, the pyrolytic carbon acquires a higher orientation via carbon atomic layer rearrangement, accompanied by microstructural evolution such as self-healing of concentric ring cracks, narrowing of the fiber/matrix interface and bridging between adjacent fibers. This microstructural evolution results in a significant decline in the mechanical properties of the composites: compressive strength, flexural strength, and shear strength decreased by approximately 60%, 68%, and 71%, respectively, while the corresponding fracture strains increased by 52%, 25%, and 19%, respectively, indicating an improvement in pseudoplasticity.

## 1. Introduction

Carbon/carbon (C/C) composites have become one of the ideal candidates for thermal structural materials in high-temperature applications [1,2,3,4], as they possess excellent properties like low density, chemical inertness, low wear, high and stable coefficient of friction, excellent thermal properties, and dimensional stability in severe environments. In recent years, C/C composites have been widely used in mechanical engineering and aerospace fields, such as hot press molds, high temperature fasteners, rocket motor nozzles in aerospace [5,6,7], and so on. The development of modern composite materials requires a profound understanding of friction and wear mechanisms, particularly in power transmission elements where maximum efficiency is sought. Performance optimization often depends on the microstructural organization and the addition of specific nanocomposite additives that can modify the properties of the base material [8].

The mechanical properties of C/C composites are known to be strongly governed by their microstructure, which encompasses factors such as pyrolytic carbon (PyC) texture, carbon fiber type, fiber/matrix interface characteristics, and the distribution of matrix defects. According to the classification proposed in the literature, PyC matrices produced via CVI are typically categorized as smooth laminar (SL), rough laminar (RL), or isotropic (ISO), with SL exhibiting superior strength compared to the other two textures [9]. The influence of fiber type on the microstructure and resulting mechanical performance of C/C composites has also been demonstrated [10]; specifically, composites derived from carbon fiber preforms show improved mechanical properties relative to those fabricated from pre-oxidized fiber preforms. Furthermore, the incorporation of graphene sheets at the fiber/matrix interface has been shown to enhance flexural strength, interlaminar shear strength, and storage modulus in C/G/C composites when compared to conventional C/C composites without graphene [11].

High-temperature treatment (HTT), particularly at temperatures exceeding 2300 °C, has been extensively investigated as an effective means of tailoring the microstructure and mechanical properties of C/C composites [12,13,14,15,16]. Piat et al. examined how heat treatment temperature influences the mechanical behavior of tangentially fiber-reinforced composites with medium-textured (MT) and high-textured (HT) matrices, reporting that the strength of HT materials declined with rising temperature (from 2200 °C to 2900 °C) while exhibiting pseudoplastic characteristics, whereas MT materials showed a gradual strength increase accompanied by brittle behavior [17]. According to Granoff et al., heat treatment leads to the formation of distinct cracks in SL PyC, which considerably degrades the mechanical performance of C/C composites [18]. Fujita et al. investigated the effect of heat treatment temperature on the interfacial shear strength (IFSS) of C/C composites, finding that IFSS decreased during carbonization up to 1200 °C but increased upon graphitization at 2000 °C. Meanwhile, graphitization also caused matrix exfoliation from certain fibers, resulting in a very low level of shear fracture force in those samples [19]. Yu et al. [20] reported that for C/C composites with an SL PyC matrix, the flexural strength decreased as the heat treatment temperature rose from 2200 °C to 2500 °C. Similarly, Xia et al. [21] observed that heat treatment induced distinct interfacial and concentric cracks within the SL PyC, leading to a sharp reduction in flexural strength. While previous studies have primarily focused on the changes in mechanical properties and the degree of graphitization of C/C composites after heat treatment at various temperatures, there remains a lack of in-depth discussion on the underlying mechanisms of microstructural evolution during HTT.

The present study focuses on understanding how microstructural evolution influences the mechanical performance of C/C composites after HTT. To achieve this, polarized light microscopy (PLM), Raman spectroscopy, and transmission electron microscopy (TEM) are employed to characterize the texture, fiber/matrix interface, and internal defects of the composites. Furthermore, by comparing C/C composites subjected to different heat treatment temperatures, the correlation between microstructural evolution and mechanical properties is systematically explored. Unlike most existing studies, which are typically conducted at heat treatment temperatures below 2500 °C and focus primarily on the evolution of pyrolytic carbon texture, the present study investigates the evolution mechanisms of internal defects (particularly cracks and interfaces) in C/C composites at a higher heat treatment temperature of 2700 °C. This temperature corresponds to a specific engineering requirement (e.g., a critical component of a propulsion system), while the 2300 °C condition serves as the standard industrial baseline. By comparing these two temperatures, this work provides practical guidance for understanding the service performance and failure mechanisms of C/C composites under more demanding aerospace conditions.

## 2. Experimental

### 2.1. Material Preparation

**Figure 1:**
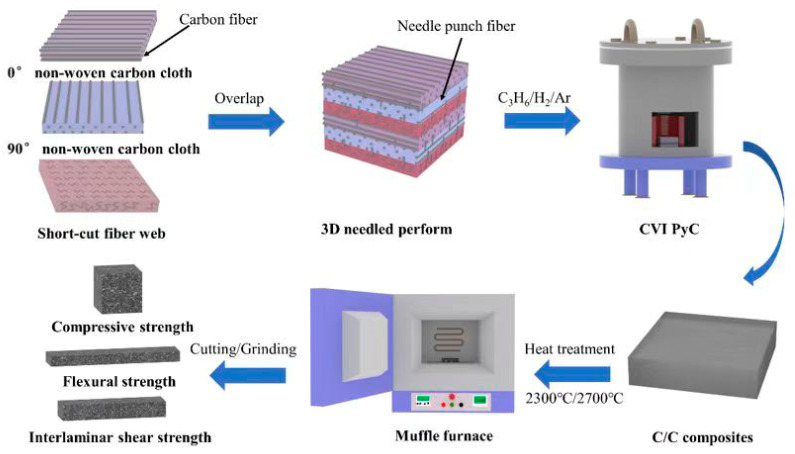
Experimental design for studying microstructural evolution and its influence on mechanical performance of heat-treated C/C composites.

A 2.5D needled PAN-based carbon fiber felt, measuring 180 mm × 180 mm × 25 mm, served as the preform. This felt was produced by alternately stacking layers of nonwoven carbon cloth and chopped fiber felt, followed by a needling process. The nonwoven cloth layers contained T300 PAN-based carbon fibers (Toray, Tokyo, Japan), while the chopped fiber felt consisted of T700 PAN-based carbon fibers (Toray). The initial density of the preform ranged from 0.42 to 0.48 g/cm^3^. Prior to densification, the felt underwent a heat treatment at 2000 °C for 2 h under an argon atmosphere to eliminate the sizing agent from the fiber surfaces. Densification was carried out in two stages: first, the density was raised to 1.60 g/cm^3^ via isothermal CVI using propylene as the carbon precursor (20 L/min) and nitrogen as the carrier and diluent gas (25 L/min); subsequently, multiple cycles of furan resin impregnation and carbonization further increased the density to 1.75 g/cm^3^. The resulting C/C composites were then subjected to final heat treatments at 2300 °C and 2700 °C in an argon atmosphere, yielding samples designated as C/C-A and C/C-B, respectively. The heating procedure was conducted as follows: the temperature was first raised to 1200 °C at a rate of 6 °C/min, then increased to 2000 °C at 4 °C/min. Above 2000 °C, the heating rate was reduced to approximately 2 °C/min. Once the target temperature was reached, the samples were held for 3 h under argon protection before being cooled to room temperature inside the furnace. A schematic illustration of the experimental design is presented in Figure 1.

### 2.2. Measurement of Mechanical Properties

An Instron 3369 universal testing machine (Instron, Norwood, MA, USA) was employed for compressive strength evaluation of the C/C composites. Specimens cut into 10 mm × 10 mm × 10 mm cubes were subjected to loading along the axis perpendicular to the friction surface, with a crosshead displacement rate set at 2 mm/min. The flexural and interlaminar shear properties were determined via three-point bending tests under identical loading orientation and displacement rate. Flexural testing utilized rectangular bars of 50 mm × 10 mm × 4 mm, supported over a span of 40 mm. The flexural strength (*σ_f_*) was derived from the equation $\sigma_{f}=(3P\times L)/(2\times \omega \times t^{2})$, where *P* maximum applied load at fracture of the samples, *L* is the span, ω and *t* are the width and the thickness of the samples, respectively. For interlaminar shear strength assessment, specimens with dimensions of 40 mm × 10 mm × 6 mm were tested using a support span of 24 mm. The shear strength (τ) was computed according to τ = 3P/(4 × b × h), in which P is the fracture load, while b and h refer to the specimen width and height, respectively. A minimum of ten specimens were tested under each condition to obtain reliable results. Post-test fracture surface observations were carried out using scanning electron microscopy (SEM, FEI Helios Nanolab G3UC, FEI Company, Hillsboro, OR, USA).

Beyond the evaluation of macroscopic mechanical properties, nanoindentation tests were conducted to determine the elastic modulus (E_IT_) and indentation hardness (H_IT_) across various microstructural constituents of the composites. A CSM Indentation Tester equipped with a Berkovich diamond indenter was employed for this purpose. The examined regions comprised the fiber core, fiber skin, as well as the PyC matrix and the PyC region adjacent to the fiber/matrix interface. Using the load–displacement curves recorded under an applied load of 4 mN, the influence of heat treatment temperature on the micromechanical behavior of the C/C composite was investigated.

### 2.3. Microstructural Investigation

The polished cross-sections of the C/C composites were examined using a polarized light microscope (Leica MeF3A, Wetzlar, Germany) to observe their morphology. Raman spectroscopic analysis was performed on a Horiba Jobin Yvon LabRam HR800 system (HORIBA, Montbonnot-Saint-Martin, France) with a 532 nm excitation laser, and spectra were acquired in the range of 1000–2000 cm^−1^. Through single-spot scanning, the textural characteristics of various microregions were identified. For microstructural investigation of the fiber/matrix interface, spherical aberration-corrected transmission electron microscopy (FEI Titan G2 60–300, er-c, Hillsboro, OR, USA) was employed, combined with selected area electron diffraction (SAED). Orientation angles (OA) were calculated on the basis of selected area electron diffraction (SAED) patterns acquired from the samples. Thin cross-sectional lamellae of the C/C composites, dedicated for transmission electron microscopy (TEM) characterization, were fabricated via the focused ion beam (FIB) milling technique with a FEI Helios Nanolab G3UC system (FEI Company, Hillsboro, OR, USA).

## 3. Results and Discussion

### 3.1. Microstructural Development in C/C Composites as a Function of HTT

**Figure 2:**
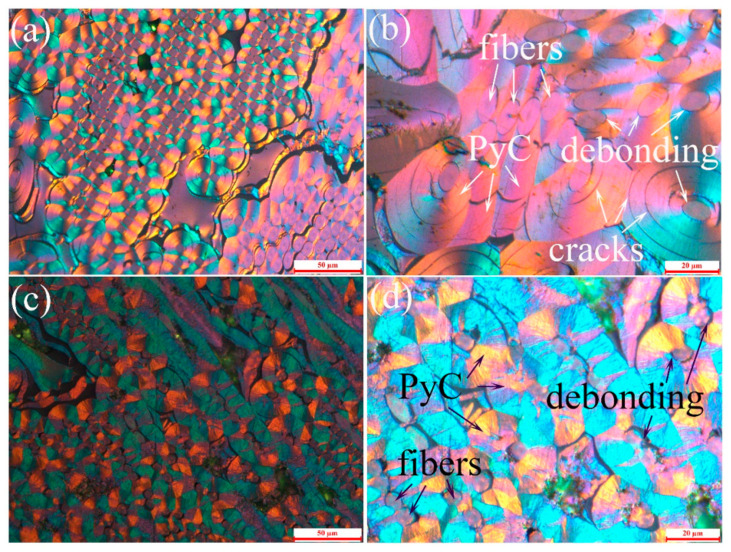
Polarized light microscopy images of the C/C composites after heat treatment: (**a**,**b**) sample C/C-A; (**c**,**d**) sample C/C-B.

Presented in Figure 2 are PLM images of the C/C composites after heat treatment at two distinct temperatures. In C/C-A (Figure 2a,b), the carbon fibers are encased by concentric PyC layers, and the PyC cross-section exhibits clear optical activity indicative of an SL texture. The relatively thick PyC layer present in the chopped fiber felt layer likely contributes to elevated thermal stresses during heat treatment. Accordingly, a greater number of concentric annular cracks are observed near the fiber/matrix interface and within the SL PyC matrix in this region (Figure 2b) compared with the nonwoven cloth layer (Figure 2a). In contrast, the PyC cross-section of C/C-B (Figure 2c,d) reveals abundant columnar growth cones that reduce its visual smoothness. Obvious microstructural disparities are observed between the C/C-B and C/C-A specimens, with three key features standing out: first, the pyrolytic carbon in C/C-B exhibits a more pronounced optical response that is typical of a rough laminar texture; second, the bonding interface between carbon fibers and PyC matrix becomes blurred, and the cross-sections of carbon fibers present an irregular round shape; third, distinct carbon-bridging structures form between adjacent fibers in the nonwoven carbon cloth layers of C/C-B (Figure 2c). A striking finding is the absence of concentric circular cracks in the PyC matrix of the C/C-B sample (Figure 2d), which is in contrast to the traditional viewpoint that elevated heat treatment temperatures would cause cracks to propagate and widen in carbon-based composites [21]. These results collectively illustrate that the 2700 °C high-temperature heat treatment not only induces the structural conversion of PyC from smooth laminar to rough laminar type, but also effectively promotes the self-repairing of intrinsic concentric circular cracks in the PyC matrix.

**Figure 3:**
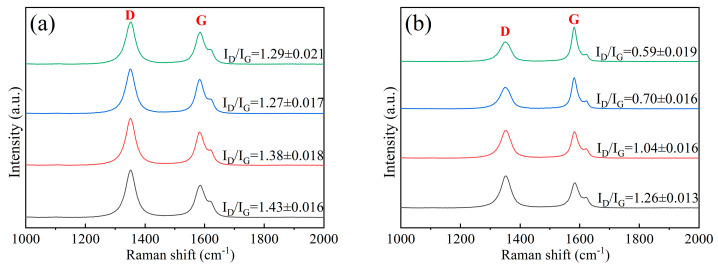
Raman spectroscopic analysis of different microstructural regions within the composites: (**a**) C/C-A; (**b**) C/C-B.

Raman spectra acquired from various microregions of C/C-A and C/C-B are presented in Figure 3. Four curves (A–D) represent four sites: fiber core, fiber skin, PyC near the fiber/matrix interface, and PyC matrix. It should be noted that the Raman data for the PyC matrix and fiber core are derived from prior work [15]. In the Raman shift range of 1000–2000 cm^−1^, all measured spectra show two typical characteristic bands—the D band and G band. These two bands are well recognized as the spectral signatures corresponding to carbon lattice defects and the degree of crystallographic ordering in carbon materials, respectively [22,23,24,25]. The graphitization degree of each microregion was quantified by the R factor (I_D_/I_G_), which is the ratio of the integrated peak intensities of the D band to the G band. To assess the statistical reliability of the I_D_/I_G_ ratios, 10 independent measurement spots were analyzed for each location. The mean I_D_/I_G_ ratios and standard deviations are annotated next to the representative spectra. The representative spectra shown in Figure 3 were selected from measurement spots with I_D_/I_G_ values closest to the respective means. In general, a smaller R value represents a higher graphitization degree, while a larger value implies lower graphitization.

As shown in Figure 3, the PyC phase in both C/C-A and C/C-B has a higher graphitization degree than the carbon fiber phase. Additionally, for the carbon fibers themselves, the skin zone exhibits a higher graphitization level than the inner core zone in both samples. With the heat treatment temperature increasing from 2300 °C to 2700 °C, the Raman spectrum of C/C-B shows a marked rise in G band intensity and a significant fall in D band intensity, in sharp contrast to those of C/C-A. These spectral variations demonstrate that the high-temperature heat treatment at 2700 °C provides sufficient thermal energy to trigger the atomic rearrangement of the carbon structure. This atomic reconfiguration in turn enhances the preferential orientation of graphite basal planes and accelerates the growth of graphite microcrystallites in the C/C-B composite.

**Figure 4:**
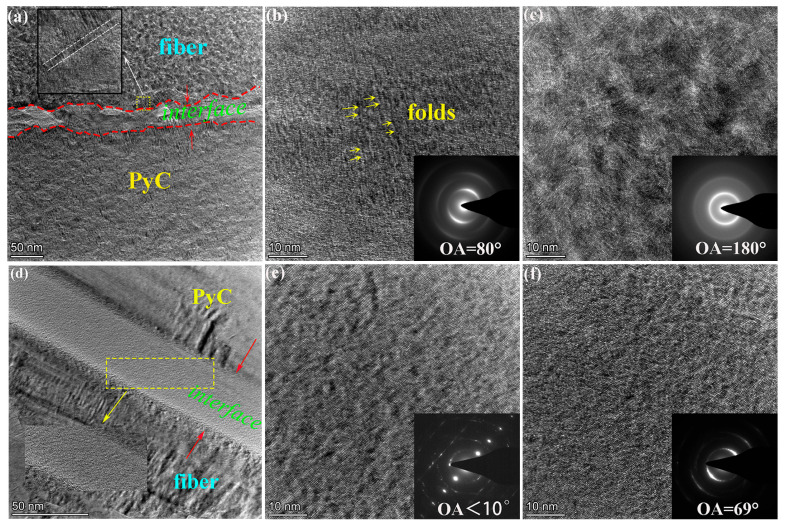
TEM micrographs of various microstructural regions in C/C-A (**a**–**c**) and C/C-B (**d**–**f**), including the interface (**a**,**d**); the PyC (**b**,**e**); the fiber (**c**,**f**).

Figure 4 shows TEM characterizations of the microstructural characteristics in different zones of the two C/C composite samples. For the C/C-A sample, the fiber-matrix interface (Figure 4a) has an irregular morphology, with an amorphous carbon interlayer of 30–55 nm in thickness formed at the interface. This amorphous carbon structure is formed due to the basal plane matching effect between carbon fibers and pyrolytic carbon. In addition, the rough surface of carbon fibers and their low graphitization degree lead to the deposition of amorphous PyC during the initial stage of the infiltration process [26]. High-resolution TEM observation of the area marked by the dashed box reveals that there are well-oriented graphene layer domains on both sides of the interface, with the thickness of these domains around 10 nm. During the high-temperature heat treatment process, thermal stress generated by the difference in thermal expansion coefficients between carbon fibers and PyC drives the preferential graphitization of the material in the interface zone [27]. On the contrary, the fiber–matrix interface of C/C-B is much straighter than that of C/C-A, and the amorphous carbon layer at its interface is significantly narrowed, with a width of only 30 nm. As shown by the HRTEM image of the marked area in Figure 4d, the size of the ordered coherent domains on both sides of the C/C-B interface is larger than that observed in the C/C-A sample. The results of HRTEM and SAED examinations of the PyC are displayed in Figure 4b,e. For C/C-A (Figure 4b), numerous elongated folded structures with a consistent orientation are observed within the PyC, indicating that the graphene layers possess some degree of orientation but remain limited in size and exhibit curvature. An OA of 80° further confirms the presence of an MT structure. In contrast, the PyC in C/C-B, which underwent heat treatment at 2700 °C, shows a substantially higher level of graphitization. The initially curled graphene layers become flattened, and the formation of interlayer bonds through dangling bonds at layer edges facilitates both interlayer bonding and lateral expansion, thereby driving microcrystalline growth. As a result, the PyC transforms from MT to an HT structure, with the OA decreasing to below 10° (Figure 4e). With respect to the carbon fibers, C/C-A exhibits a turbostratic graphite configuration characterized by atomic layers arranged in a random and uniform manner (Figure 4c). For C/C-B, the fibers demonstrate an improvement in graphitization comparable to that observed in the PyC, with an OA of 69° indicative of a medium-textured structure (Figure 4f).

**Figure 5:**
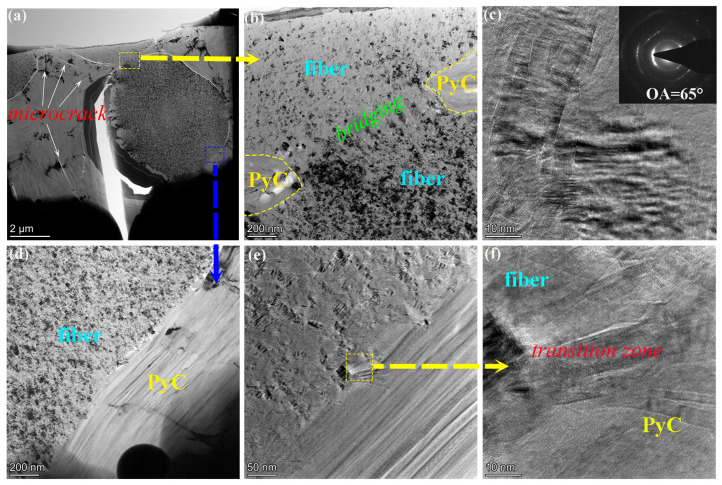
TEM micrographs of the bridging region between fibers (**a**–**c**) and the transition zone between fiber and PyC (**d**–**f**) in C/C-B.

Two main microstructural features are observed in the TEM images of C/C-B (Figure 5): a bridging region that forms between neighboring fibers, and a transition zone located at the fiber/PyC boundary. Figure 5b shows that when two carbon fibers are positioned in close proximity, a bridging zone with a width of about 1.5 μm is created. This finding is further supported by HRTEM examination of the area (Figure 5c). The bridging zone contains numerous folded structures that are all oriented in the same direction. The presence of such uniformly aligned folds indicates that the graphene lamellae in this zone are preferentially oriented. The measured OA of 65° confirms that this area possesses a medium texture. Figure 5d and e reveal the existence of a transitional layer between the fiber and the surrounding PyC. Unlike the amorphous carbon that constitutes the conventional fiber/matrix interface, the graphene layers within this transitional region display a noticeable degree of preferred orientation, as demonstrated in the HRTEM image of Figure 5f. It is worth noting that although concentric annular cracks are no longer visible in C/C-B under PLM after treatment at 2700 °C, TEM examination (Figure 5a) reveals that numerous microcracks remain present within the PyC. This inconsistency can be explained by the dimensional changes that occur during high-temperature graphitization [28]. These dimensional changes help to close the concentric annular cracks, yet they do not completely eliminate them at the microscopic scale.

**Figure 6:**
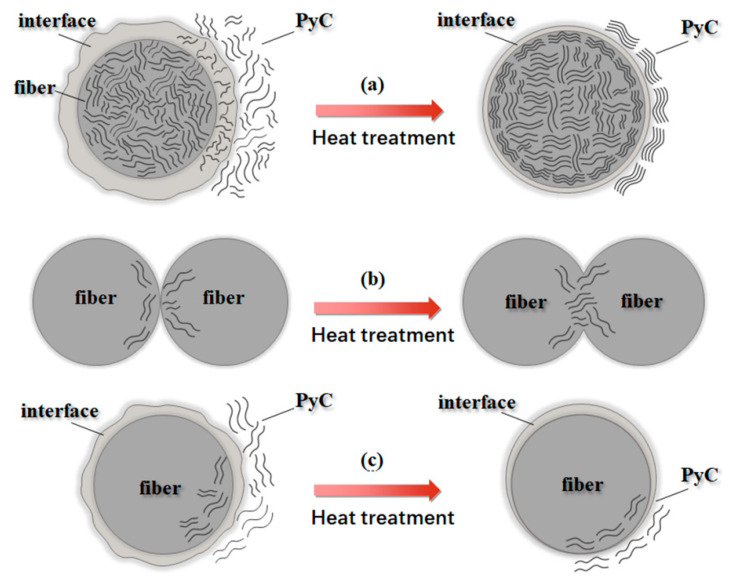
Schematic illustration of microstructural changes before and after heated: (**a**) fiber/PyC interface, (**b**) bridging region between fibers, (**c**) transition zone between fiber and PyC.

A schematic overview of how the PyC matrix and fibers evolve microstructurally during heat treatment is presented in Figure 6. Figure 6a shows that the fiber/PyC interface exhibits a dynamic, sandwich-like structure during heat treatment. Under the influence of stress-induced graphitization, atomic rearrangement of carbon atoms occurs preferentially at the edges of the interfacial layer, then gradually diffuses inward. The same behavior is observed at both the carbon fiber and the PyC matrix sides.

In the TEM images, this is manifested as a thinning of the interfacial layer after the 2700 °C heat treatment. Figure 6b clearly illustrates the formation of bridging structures between two adjacent carbon fibers during high-temperature treatment. This phenomenon can be divided into two stages. First, carbon atoms acquire sufficient energy to drive the transformation from the original metastable turbostratic structure to a more stable three-dimensional ordered graphitic structure. When this process reaches a certain extent, adjacent carbon crystallites with similar orientations gradually align and merge, eliminating the boundaries and defects between them. This results in the inter-fiber bridging observed in the study. Figure 6c demonstrates that the formation of a transition zone and the straightening of the fiber/PyC interface are jointly driven by surface energy minimization and atomic diffusion. Under high temperature, atoms at the originally irregular interface gain enough energy to rearrange, ultimately tending toward a straighter, more stable state with a smaller total surface area.

### 3.2. Mechanical Performance

**Figure 7:**
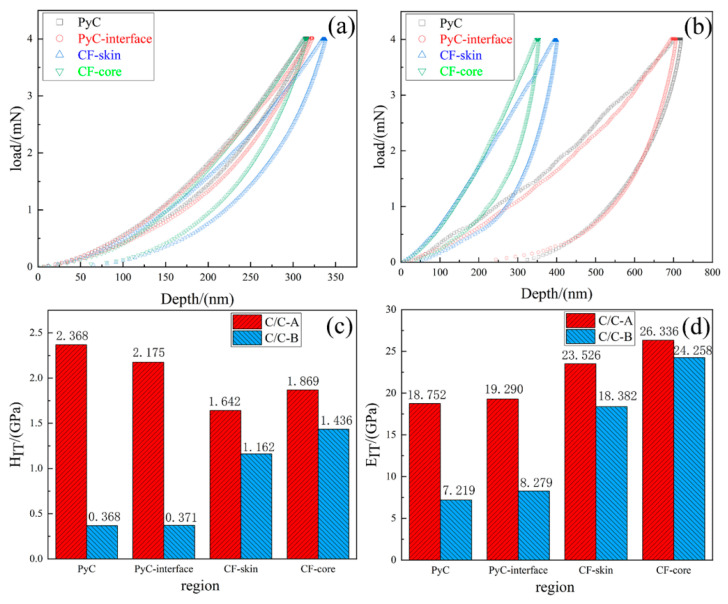
Nanoindentation results of C/C-A and C/C-B across different microregions: (**a**,**b**) load-depth curves; (**c**) indentation hardness; (**d**) indentation modulus.

The mechanical response of carbon fibers and PyC at the microscale was assessed through nanoindentation experiments conducted at four distinct locations within the composites: the bulk PyC matrix, the PyC layer adjacent to the fiber/matrix boundary, and the outer skin and inner core regions of the carbon fibers. A comparison of the load versus displacement profiles for C/C-A and C/C-B (Figure 7a,b) alongside the derived HIT and EIT values (Figure 7c,d) shows that both constituent phases in C/C-B possess markedly reduced mechanical performance relative to those in C/C-A. The most dramatic difference occurs in the PyC phase, where HIT drops from 2.368 GPa to 0.368 GPa and EIT decreases from 18.752 GPa to 7.219 GPa. Such property variations are consistent with the microstructural transformations identified through TEM and Raman analyses. Subjecting the material to 2700 °C promotes the development of more perfect graphite crystallites and a more strongly preferred orientation within C/C-B, where the graphene layers consist largely of sp^2^-hybridized carbon bonded via weak van der Waals interactions [29,30,31]. Earlier findings by Lieberman and Pierson [32] indicated that PyC with a high-texture character is intrinsically softer than its medium-texture counterpart. This phenomenon arises because in medium-texture PyC, the carbon layers adopt a curled configuration and become densely interwoven, creating considerable resistance as the indenter penetrates the matrix. By contrast, in high-texture PyC, the layers are more perfectly aligned, enabling easier indenter penetration with reduced resistance from interlayer slip. As a result, the measured HIT and EIT values are consistently higher for C/C-A than for C/C-B. A similar trend is observed when comparing different microstructural regions within the same composite.

**Figure 8:**
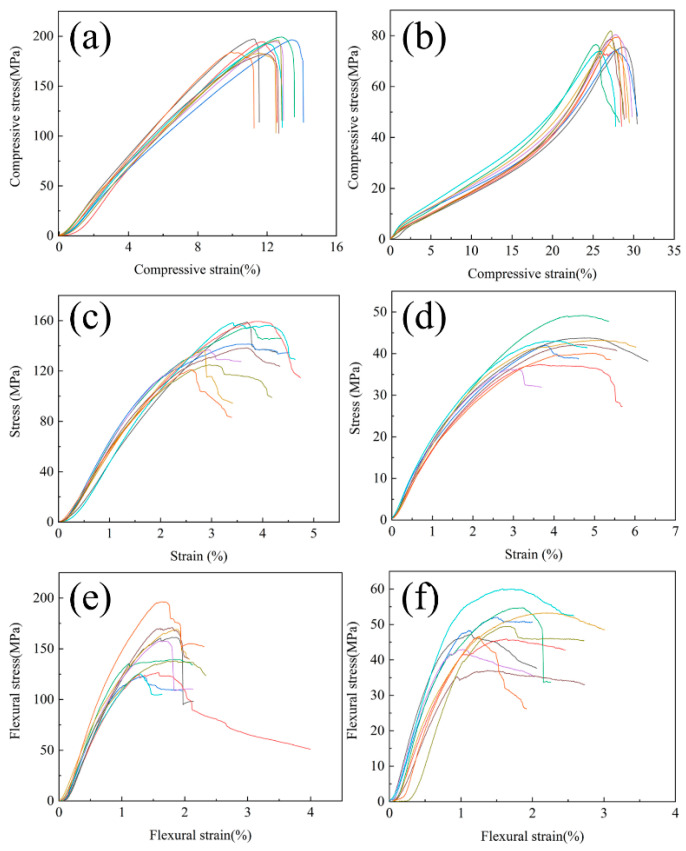
Mechanical testing curves of the C/C composites under different loading conditions: compressive test results (**a**,**b**), interlaminar shear test results (**c**,**d**), and flexural test results (**e**,**f**). The left column (**a**,**c**,**e**) corresponds to sample C/C-A, while the right column (**b**,**d**,**f**) corresponds to sample C/C-B.

Figure 8 presents the mechanical test curves of C/C-A and C/C-B, with their mechanical properties summarized in Table 1. Based on compressive test curves of both composites depicted in Figure 8a,b, it is evident that both materials exhibit nearly linear elastic behavior prior to reaching their respective maximum loads. Subsequently, C/C-B demonstrates a near-parabolic decline in its stress–strain curve, whereas C/C-A exhibits a vertical decline. Furthermore, the rupture strain of C/C-B is twice that of C/C-A. These findings suggest that C/C-B possesses superior deformation capacity.

The interlaminar shear and flexural behaviors of the two composites are presented in Figure 8c,d and Figure 8e,f, respectively. Examination of the stress–strain curves for C/C-A (Figure 8c,e) reveals that the stress increases approximately linearly with strain during the early loading stage. After reaching the peak value, the stress declines in a stepwise or zigzag manner, which is characteristic of pseudoplastic fracture behavior [33,34,35,36]. In the case of C/C-B, the post-peak stress reduction follows a more gradual, approximately parabolic path (Figure 8d,f). This less abrupt decline in stress after the maximum point suggests that C/C-B possesses greater pseudoplasticity than C/C-A, which aligns with the higher failure strain values recorded for this material.

To further evaluate the energy absorption capacity of the two materials, the volumetric fracture energy (toughness) was calculated by integrating the area under each stress–strain curve in Figure 8 from zero strain to the fracture point. The calculated mean values for C/C-A are 1246 MJ/m^3^ (compression), 236 MJ/m^3^ (shear), and 181 MJ/m^3^ (bending). For C/C-B, the corresponding values are 825 MJ/m^3^ (compression), 145 MJ/m^3^ (shear), and 177 MJ/m^3^ (bending). Compared to C/C-A, C/C-B exhibits a reduction of approximately 33.8% in compressive fracture energy, 38.6% in shear fracture energy, and only 2.2% in bending fracture energy. The bending strength decreased by approximately 67%, while the toughness remained nearly unchanged, indicating that the high-temperature heat treatment significantly improved the deformability of C/C-B under bending conditions.

According to the data presented in Table 1, it can be observed that the mechanical properties of C/C-B experienced a significant decline of around 70% when subjected to heat treatment at a temperature of 2700 °C, in comparison to the corresponding properties of C/C-A. Several factors account for the decline in mechanical properties. First, while high-temperature graphitization promotes graphite microcrystal growth and texture ordering, it simultaneously reduces the mechanical properties of PyC and fibers—the primary cause of the observed decline [30,31]. Second, fiber-matrix debonding and interface weakening occur during HTT [37], along with bridging between adjacent carbon fibers. These effects collectively compromise load transfer within the composite, thereby reducing its overall mechanical performance.

**Figure 9:**
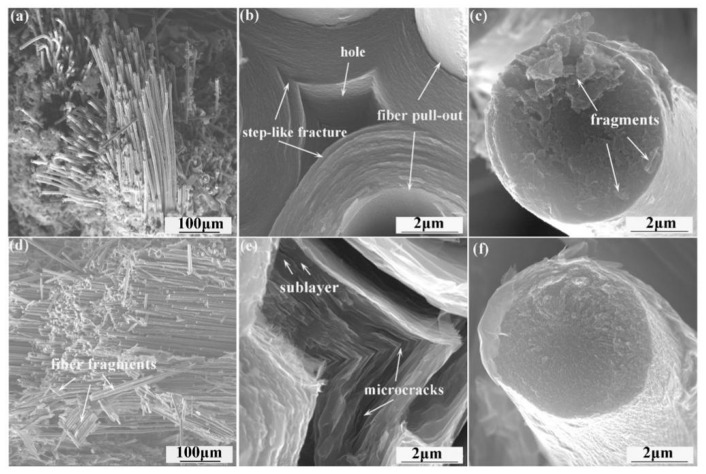
Representative SEM micrographs of the fractured cross-sections obtained from flexural test specimens: panels (**a**–**c**) correspond to C/C-A, while panels (**d**–**f**) correspond to C/C-B.

Figure 9 presents SEM images of the fracture surfaces obtained from specimens after flexural testing. Examination of Figure 9a,d reveals that both composites exhibit fracture surfaces featuring numerous intact fiber bundles with varying pullout lengths. This morphology indicates that crack propagation occurred preferentially along the fiber/matrix interface rather than directly through the carbon fibers themselves, resulting in debonding of the fibers from the surrounding matrix. Consequently, the fibers were pulled out and ultimately fractured. This mechanism resulted in the absorption of a substantial amount of energy, which is the main reason for the pseudoplastic behavior exhibited by the C/C composites upon fracture and failure. It can also be seen that the fracture surface of C/C-B contains more fiber fragments than that of C/C-A, which may be related to the lower mechanical strength of the carbon fibers as well as the fusion between the fibers in C/C- B. Meanwhile, Figure 9b reveals that the PyC of C/C-A exhibits a relatively flat section, but the carbon fiber section displays the presence of varying sizes of debris. Notably, the fiber’s skin region exhibits a higher concentration of debris, while the core region seems comparatively smooth. This may be due to the higher graphitization in the skin region, where the graphene layers slip as the crack propagates, eventually leading to the formation of flake debris during fracture. Conversely, the core region exhibits a fracture mode resembling brittle fracture, characterized by a smoother cross-section and less debris. This distinction can be attributed to the ‘turbostratic graphite structure’ present in the core region, which results in a higher modulus and stronger brittle fracture characteristics (as shown in Figure 9c).

Figure 9e illustrates that within C/C-B, the PyC is composed of sublayers with thickness at the nanometer scale, and a large number of microcracks exist between these sublayers. This particular microstructure facilitates interlayer slip of the graphene sheets when the material is subjected to mechanical loading, thereby causing cracks to deviate as they propagate through the matrix. As a result, the speed at which cracks advance is slowed, giving rise to more pronounced pseudoplastic fracture behavior and increased strain at failure. In addition, a comparison of Figure 9f with the corresponding images of C/C-A reveals that the wear debris on the fiber sections of C/C-B is both finer and more uniformly dispersed. This observation can be explained by the elevated graphitization level achieved in C/C-B following the higher temperature treatment.

### 3.3. Fracture Failure Analysis on C/C Composites

**Figure 10:**
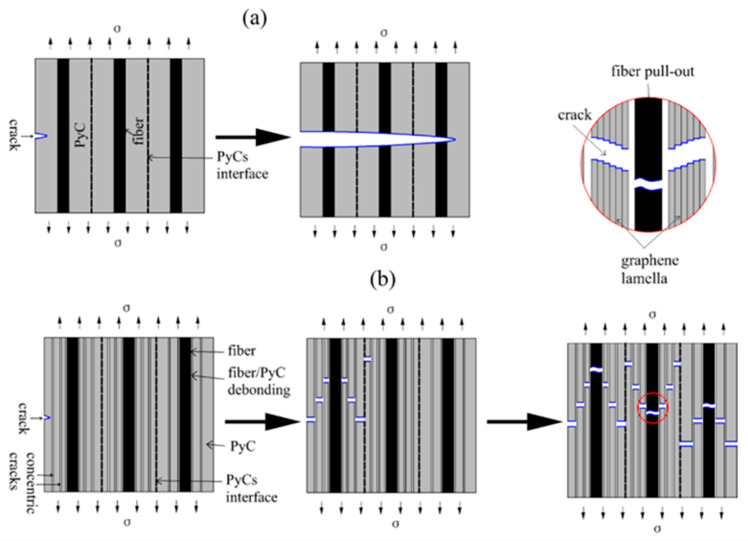
Schematic diagram of the fracture mechanism of C/C composites. (**a**) C/C-A; (**b**) C/C-B.

Figure 10 schematically illustrates the fracture mechanism of C/C composites with different microstructures. In C/C-A, numerous concentric ring cracks in the pyrolytic carbon deflect propagating cracks upon encountering the matrix cracks. As cracks reach the fiber/matrix interface, they are blocked and cannot cut directly through the fiber, and so preferentially expand forward along the fiber/matrix interface, bypassing the fibers, resulting in a sequence of behaviors such as the fiber/matrix interface debonding, bending, fiber pull-out, fracture, etc. Eventually a large number of fibers can be seen on the cross-section of the specimen. The surface of the extracted carbon fibers is smooth, and the PyC matrix near the root of the extracted fibers is still relatively intact, with the appearance of a “step-like” or “canine” (Figure 9a,b). As a large number of fibers or bundles of fibers in the composite are extracted from the matrix during the fracture process, the fracture toughness of the material is improved. the fracture characteristics of C/C-A exhibit some pseudoplasticity (Figure 8e).

Notably, despite exhibiting lower mechanical strength than C/C-A, C/C-B demonstrates enhanced deformability. This is evidenced by the stress–strain behavior shown in Figure 8f, where larger failure strains are observed and the post-peak stress follows a trajectory closer to a parabolic shape. Such behavior can be linked to the presence of highly textured PyC within C/C-B. As illustrated in Figure 9e, this type of PyC is characterized by numerous sublayers with thickness at the nanometer scale. When cracks propagate through the matrix, these sublayers act as barriers that deflect the crack path. In addition, the ability of graphene layers to undergo shear slip under loading further contributes to the pseudoplastic nature of the C/C composites.

## 4. Conclusions

This work systematically investigates the evolution of microstructures and the corresponding changes in mechanical behaviors of C/C composites subjected to high-temperature heat treatment at 2300 °C and 2700 °C, and the key conclusions are summarized as follows.

Microstructural evolution:

Raising the heat treatment temperature to 2700 °C drives the transformation of the pyrolytic carbon matrix from a smooth laminar texture to a rough laminar one. Intrinsic concentric annular cracks in the matrix are effectively closed, which is contrary to previously reported observations. Thermal-induced atomic rearrangement refines the fiber/PyC interface into a flatter and thinner structure. Carbon-bridging structures form between adjacent carbon fibers, a phenomenon not documented in prior studies.

Mechanical performance:

The C/C composite treated at 2700 °C exhibits significant degradation in both strength and elastic modulus. In contrast, the material’s deformability is remarkably improved.

Structure-property relationship:

The trade-off between mechanical strength and deformability is mainly attributed to the high graphitization degree and the weakened interfacial bonding strength between carbon fibers and the PyC matrix.

Comparison with hypotheses and literature:

The experimental outcomes largely confirm the expected trends (graphitization, interfacial smoothing, strength-deformability trade-off). Two unexpected findings deviate from existing literature: (a) complete closure of intrinsic cracks, and (b) formation of carbon-bridging structures.

These observations challenge conventional understanding and offer new perspectives on the high-temperature behavior of C/C composites.

## Figures and Tables

**Table 1:** Mechanical performance parameters of the C/C composite specimens.

Specimen	Compressive Strength (MPa)	Interlaminar Shear Strength (MPa)	Flexural Strength (MPa)
A	191.70 ± 6.21	17.88 ± 1.77	150.78 ± 24.65
B	77.1 ± 2.79	5.25 ± 0.45	48.94 ± 6.45

## Data Availability

The original contributions presented in this study are included in the article. Further inquiries can be directed to the corresponding author.
